# N-Acetylcysteine Amide Is a Potential Novel Radioprotector of Salivary Gland Function

**DOI:** 10.3390/cancers17172902

**Published:** 2025-09-04

**Authors:** Amit Ritter, Elad Hikri, Hongyan Li, Ela Markovsky, Gideon Bachar, Noga Kurman, Aron Popovtzer, Adriana Haimovitz-Friedman, Aviram Mizrachi

**Affiliations:** 1 Department of Otolaryngology Head and Neck Surgery, Rabin Medical Center, Petah Tikva 4941492, Israel; 2 Gray Faculty of Medical & Health Sciences, Tel-Aviv University, Tel Aviv 6997801, Israel; 3 Department of Radiation Oncology, Memorial Sloan Kettering Cancer Center, 1275 York Avenue, New York, NY 10065, USA; 4 Davidoff Cancer Center, Rabin Medical Center, Petah Tikva 4941492, Israel; 5 Department of Oncology, Hadassah-Hebrew University Medical Center, Jerusalem 9112102, Israel

**Keywords:** acetylcysteine, head and neck neoplasms, radiation, salivary gland diseases, xerostomia

## Abstract

**Background:** Radiation-induced salivary gland (SG) hypofunction is mediated via microvascular dysfunction and radical oxygen species. N-acetylcysteine amide (NACA) has shown antioxidant properties with low toxicity. We explored NACA’s potential as a radioprotector of SG function. **Methods:** Bovine aortic endothelial cells (BAECs) were treated with NACA before irradiation with a single 10 Gy dose. Apoptosis was assessed by bis-benzimide staining and quantified via fluorescence microscopy. In vivo, NACA was administered to mice prior to a single 15 Gy head and neck irradiation. Eight weeks post-irradiation, saliva production was measured using pilocarpine stimulation; lysozyme levels were analyzed by ELISA. SGs were collected for immunohistochemistry. **Results:** BAEC apoptosis was substantially lower in NACA-treated cells vs. radiation-only (10% vs. 23%). In vivo, mice lost significant weight and developed severe hair loss eight weeks post-irradiation—attenuated by NACA pretreatment. Saliva production was reduced by 72% post-radiation, with a corresponding drop in lysozyme. NACA increased salivary flow by 42% and prevented lysozyme reduction. Post-radiation decline in microvessel density was also prevented by NACA. **Conclusions:** These outcomes suggest NACA may serve as a radioprotector of SG function in patients undergoing radiotherapy for head and neck cancer.

## Simple Summary

This study explores the potential of N-acetylcysteine amide (NACA) to protect salivary gland function from radiation-induced damage. Radiation impairs salivary glands through microvascular injury and oxidative stress. In cell culture, NACA reduced radiation-induced endothelial cell death. In a mouse model, NACA was administered before head and neck irradiation. Treated mice showed reduced weight loss, hair loss, and salivary dysfunction. NACA preserved saliva production, maintained lysozyme levels, and prevented microvessel loss in the glands. These findings suggest NACA may be an effective, low-toxicity radioprotective agent for salivary gland preservation during head and neck cancer treatment.

## 1. Introduction

Radiation therapy (RT) has a major role in the curative treatment of head and neck squamous cell carcinomas (HNSCC). Chemotherapy may be added for radiosensitization in cases of advanced-stage cancers and recurrent diseases [1]. One of the most common side effects of RT is salivary gland (SG) injury. Damage to both major and minor SGs causes salivary hypofunction in approximately 80% of patients and may eventually lead to permanent xerostomia [2]. The resulting morbidity, including dysphagia, mucosal infections, reduced nutrition intake, and speech deficiencies, adversely reduces patients’ quality of life [3,4]. These complications are often long-lasting and difficult to manage, and they remain one of the major factors impacting survivorship experience in head and neck cancer patients.

The underlying mechanism of RT-induced SG damage has been considered enigmatic since it was first described more than 100 years ago [5]. Early studies focused on the early and delayed phases of DNA damage after RT, resulting in acinar progenitor cell death [6,7]. However, more recent evidence suggests that the primary target of radiation within the SGs may not be the epithelial cells alone. It has been found that microvascular endothelial cells within the SGs are highly sensitive to radiation and are likely critical in initiating the damage cascade [8]. Radiation injury to endothelial cells may result in both acute and delayed tissue damage, contributing to long-term gland dysfunction [9]. Molecular studies have shown that this endothelial injury involves specific signaling pathways originating at the cell membrane, most notably the acid sphingomyelinase/ceramide (ASMase/Ceramide) pathway [10,11]. Activation of this pathway results in the generation of ceramide and reactive oxygen species (ROS), which in turn lead to endothelial apoptosis and vascular dysfunction. Our preliminary studies have demonstrated that RT-induced SG hypofunction is mediated via this mechanism of endothelial apoptosis and microvascular disruption involving ceramide accumulation and ROS generation [12].

Current management approaches for SG hypofunction remain largely palliative and are generally unsatisfactory. Saliva substitutes and sialogogues provide limited and temporary relief. Amifostine (WR-2721; 2-[(3-aminopropyl) amino] ethylphosphorothioic acid), originally developed to reduce nephrotoxicity associated with platinum-based chemotherapy, has been approved for prevention of xerostomia in patients undergoing RT for head and neck cancer. However, its use remains limited due to significant side effects, the need for intravenous administration, and ongoing debate about potential tumor protection [13].

A recently discovered compound, N-acetylcysteine amide (NACA), the amide form of N-acetylcysteine (NAC), has shown promising antioxidant properties with significantly improved bioavailability and a very low toxicity profile. Unlike NAC, NACA is lipophilic and is able to cross cellular membranes and the blood–brain barrier. It can scavenge free radicals, chelate metal ions such as copper, and protect red blood cells from oxidative damage [14]. Preclinical studies have shown that NACA protects against methamphetamine-induced oxidative stress and neurotoxicity in human brain endothelial cells [15], and it also provides neuroprotection following traumatic brain and spinal injuries [16]. Pharmacokinetically, NACA achieves higher plasma concentrations than NAC, with oral bioavailability around 67% in mice—over four times greater than NAC under similar conditions [17]. It is rapidly absorbed, converted to NAC, and reaches plasma levels in the 50 ng/mL to 50 μg/mL range [18]. Its enhanced tissue penetration and three- to fourfold greater glutathione-replenishing capacity further support its therapeutic potential [17].

Based on these findings and its favorable pharmacological characteristics, we sought to explore the potential role of NACA as a radioprotective agent for preserving SG function during RT. In this study, we demonstrate that pretreatment with NACA effectively reduces RT-induced endothelial apoptosis in vitro in a bovine aortic endothelial cell model and preserves salivary flow, lysozyme secretion, and vascular integrity in vivo in a murine head-and-neck irradiation model. These findings support NACA as a promising candidate radioprotector of salivary gland function.

## 2. Materials and Methods

### 2.1. In Vitro Studies

#### 2.1.1. Cell Culture and Irradiation

Bovine aortic endothelial cells (BAECs) were grown in Dulbecco’s Modified Eagle Medium (DMEM) supplemented with glucose (1 g/L), bovine calf serum (5%), penicillin (100 U/mL), streptomycin (100 µg/L), and L-glutamine (2 mM at 37 °C in a humidified 5% CO_2_ chamber. Cell culture conditions were maintained under strict aseptic technique, and all medium components were freshly prepared and sterile-filtered prior to use. Cell confluence was monitored microscopically, and only cultures at 80–90% confluence were used for irradiation experiments to ensure consistency across experimental groups.

For experiments, BAECs were starved overnight (16–18 h) in serum-free DMEM containing 0.2% human albumin to reduce basal signaling and oxidative activity, thereby allowing clearer assessment of radiation-induced responses. Cells were gamma irradiated on ice with single doses between 0 and 20 Gy using the Biobeam 8000 gamma irradiator with a Cesium-137 source (Gamma-Service Medical GmbH, Leipzig, Germany) at a rate of 2.08 Gy/min. The ice-cold irradiation setup was intended to minimize cellular metabolic activity during exposure, thereby isolating the direct effects of radiation from secondary metabolic responses. For experiments involving incubations of under 10 min, cells were kept on ice during irradiation and subsequently warmed in a 37 °C water bath for the indicated durations. This step ensured synchronized reactivation of metabolism and signaling pathways for precise temporal analysis. In the control experiments, cells were sham-irradiated and handled identically. Immediately following irradiation, all cells were incubated at 37 °C under standard culture conditions.

Stock solutions of NACA (Sigma-Aldrich, Burlington, MA, USA) and the non-specific pan-NOX inhibitor diphenyleneiodonium chloride (DPI) (Sigma-Aldrich, Burlington, MA, USA) [19] were prepared and diluted in a sterile fashion immediately prior to use to ensure compound stability. For NACA, a 1 mM concentration was selected based on previous dose–response publications [14]. For all treatment experiments, NACA (1 mM) or DPI (10 µM) was added to the cells 10 min prior to irradiation. The chosen pretreatment time of 10 min was based on preliminary optimization studies aimed at achieving adequate intracellular uptake while avoiding toxicity. Both substances were removed 1 h or 8 h later to evaluate their immediate and sustained effects, respectively. Parallel control wells treated with vehicle alone were included to rule out any nonspecific solvent effects.

An additional dose–response study was performed to evaluate the protective effects of NACA against increasing doses of RT in BAECs. Using the previously described methods, cells were pretreated with 1 mM NACA before receiving graded doses of ionizing radiation (5 Gy, 10 Gy, and 20 Gy). Apoptosis was quantified at a defined time point post-irradiation, and the resulting dose–response data were analyzed to determine the extent of inhibition at each RT dose. A parallel comparison with a higher concentration of 5 mM NACA was also conducted.

#### 2.1.2. Quantification of Endothelial Apoptosis

Morphological changes in the nuclear chromatin of BAEC undergoing apoptosis were assessed using the DNA-binding fluorochrome bis-benzimide trihydrochloride (Hoechst-33258, Sigma-Aldrich, Burlington, MA, USA), as described previously [9]. Eight hours after irradiation, BAECs were fixed with 4% paraformaldehyde (PFA) to preserve cellular structure, then washed with phosphate-buffered saline (PBS) to remove residual fixative. Cells were subsequently stained with 50 µL of bis-benzimide trihydrochloride solution, prepared by diluting 24 µL of Hoechst-33258 stock solution (10 mg/mL) into 10 mL of PBS immediately prior to use to ensure dye stability. Staining was performed for 10 min at room temperature, protected from light to prevent photobleaching. Apoptotic nuclei were quantified using a Zeiss Axioimager Z-2 fluorescence microscope (Carl Zeiss Microscopy GmbH, Jena, Germany) equipped with filters suitable for Hoechst detection. For each data point, 300 cells were counted in triplicate to ensure statistical reliability and minimize sampling bias.

### 2.2. In Vivo Studies

#### 2.2.1. RT-Induced SG Injury in an Animal Model

Female C3H mice were utilized in this study as an established in vivo model. All experiments were conducted in healthy, non–tumor-bearing mice to isolate and quantify the effects of radiation and radioprotection on salivary tissue. The animals selected were between 7 and 9 weeks of age. Their body weights ranged from 16 to 23 g, a parameter carefully monitored to ensure consistency across experimental groups and to avoid potential variability related to body mass or age. All animal procedures were conducted under a protocol that received formal approval from the Institutional Animal Care and Use Committees (IACUC) at both Tel-Aviv University and Memorial Sloan Kettering Cancer Center. The experimental design fully complied with the ethical standards and technical guidelines outlined in the Guide for the Care and Use of Laboratory Animals, issued by the National Research Council, to ensure humane treatment and scientific integrity throughout the study.

Targeted irradiation of the SGs was performed by delivering localized radiation to the head of each animal. A single dose of 15 Gy was administered specifically to the cranial region using a Therapax DXT300 X-ray irradiator (Pantak Inc., East Haven, CT, USA). The irradiator was equipped with a 2.0 mm aluminum filter and operated at 300 kVp, delivering the dose at a controlled rate of 1.9 Gy per minute. This irradiation protocol was based on previously published and validated methodologies [20]. The use of a focused head-only exposure approach enabled assessment of localized radiation effects while minimizing systemic radiation toxicity.

To evaluate the efficacy of a potential radioprotective intervention, a total of 15 mice were randomly assigned to three groups: sham-irradiated controls (N = 5), head-and-neck-irradiated without treatment (N = 5), and head-and-neck-irradiated with NACA pretreatment (N = 5). NACA was delivered intraperitoneally at a dose of 350 mg/kg, adapted from previous studies [21], precisely 10 min prior to the initiation of irradiation to allow for systemic absorption and distribution. This timing was selected based on prior pharmacokinetic data suggesting peak bioavailability within minutes of administration. Following radiation exposure, all animals were returned to standard housing conditions within the animal facility. The environment was climate-controlled and maintained on a consistent light-dark cycle to reduce physiological stress. Animals had unrestricted access to food and water throughout the recovery period. This post-treatment care regimen was implemented to ensure animal welfare and to eliminate extraneous variables that might influence the study outcomes related to tissue damage, recovery, or response to treatment.

#### 2.2.2. Evaluation of Long-Term Effects of Radiation on SG Microvasculature

*Saliva sample collection*—To evaluate the long-term impact of radiation on salivary gland function, salivary flow rates were assessed eight weeks following irradiation using a pilocarpine-induced stimulation assay. This time point was selected to allow for the development of chronic radiation-induced changes within the salivary glands, including fibrosis, acinar cell loss, and microvascular damage. Prior to sample collection, mice were weighed individually to ensure accurate dosing and to monitor any potential weight loss that might indicate systemic effects of radiation. Subsequently, light anesthesia was induced using a solution containing ketamine (100 mg/kg) and xylazine (10 mg/kg) dissolved in sterile saline. The anesthetic was administered via intraperitoneal injection to achieve a consistent and rapid onset of sedation while minimizing animal stress. Stimulation of saliva secretion was achieved by a subcutaneous injection of pilocarpine, a muscarinic receptor agonist known to robustly induce exocrine gland activity. Pilocarpine was administered at a dosage of 1 µL per gram of body weight, using a concentrated solution of 50 mg/mL. Saliva collection commenced within 2 min of pilocarpine administration to capture peak salivary response and ensure uniformity across samples. During the collection process, each mouse was positioned with care, and a 75 mm hematocrit capillary tube (Drummond Scientific Co., Broomall, PA, USA) was inserted gently into the oral cavity. Whole saliva was collected continuously for a 10 min period into pre-weighed 0.75 mL Eppendorf tubes, ensuring accuracy in volume assessment. The volume of saliva secreted was determined gravimetrically by re-weighing the tubes post-collection and calculating the difference from the pre-weighed baseline. This method provided a reliable and quantitative measure of residual salivary gland function. Following the collection procedure, and while still under the influence of anesthesia, mice were humanely euthanized by cervical dislocation in accordance with approved IACUC protocols.

*Saliva lysozyme levels*—In addition to measuring total saliva output, the quality of saliva was also assessed through analysis of lysozyme content. Lysozyme, a naturally occurring antimicrobial enzyme present in saliva, plays a critical role in oral immunity and mucosal defense. Its concentration serves as an indicator of the functional secretory capacity of the salivary glands. Saliva samples previously collected from the mice at the 8-week time point were preserved at –80 °C to prevent enzymatic degradation and maintain sample integrity prior to biochemical analysis. Lysozyme levels were quantified using a commercially available mouse-specific enzyme-linked immunosorbent assay (ELISA) kit (LifeSpan BioSciences Inc., Seattle, WA, USA), following the manufacturer’s instructions. The assay relies on antigen–antibody binding and fluorescent signal detection for accurate quantification. Measurements were taken using a fluorescent plate reader, and final concentrations were calculated after subtracting background absorbance values to correct for non-specific signal. This analysis allowed for the comparison of enzymatic content across experimental groups, providing insight into radiation-induced changes in salivary composition.

*Immunofluorescence microvessel density (MVD) assay*—To assess radiation-induced damage to the vascular component of the salivary glands, immunofluorescence-based evaluation of MVD was performed. At the conclusion of the experiment, bilateral submandibular and parotid salivary glands were carefully excised from each mouse immediately post-mortem. Tissues were fixed in 4% paraformaldehyde to preserve cellular and structural morphology, followed by paraffin embedding to facilitate histological sectioning. Tissue blocks were cut into 5 µm-thick sections using a microtome, and the slides were prepared for subsequent staining procedures. Sections underwent standard hematoxylin and eosin (H&E) staining for general histopathological evaluation. In addition, specific immunostaining was performed using primary antibodies against CD31, a marker of endothelial cells used to visualize blood vessels, and cleaved caspase-3, an apoptotic marker indicative of cell death. For quantitative MVD assessment, slides stained with anti-CD31 antibodies were selected, with 12 samples analyzed per experimental group to ensure statistical robustness. Images of stained sections were captured using a fluorescence microscope, and vessel density was calculated using the ImageJ software suite (Version 1.53c, National Institutes of Health, Bethesda, MD, USA). Automated or semi-automated image analysis workflows were applied to quantify the number of CD31-positive microvessels per unit area of glandular tissue, facilitating objective comparison between irradiated and control groups.

### 2.3. Statistical Analysis

All statistical analyses were conducted using SPSS version 23 (IBM Corp., Armonk, NY, USA) and GraphPad Prism version 6.0 (GraphPad Software Inc., La Jolla, CA, USA). Data are presented as mean ± standard deviation (SD) or mean ± standard error (SE). Prior to analysis, data distribution and variance homogeneity were assessed where appropriate. To compare two independent groups, the independent samples t-test was applied. When assumptions of equal variance were violated, Welch’s *t*-test was used instead. For comparisons involving more than two groups, one-way analysis of variance (ANOVA) was used, followed by Bonferroni correction for post hoc multiple comparisons to control for Type I error. All statistical tests were two-tailed, and a *p*-value < 0.05 was considered statistically significant for all comparisons, except when adjusted for multiple testing or specific test requirements.

Sample size and power calculations were conducted using G\*Power 3.1.9.7 (Heinrich Heine Universität Düsseldorf, Germany). Group size (N = 5) was chosen from pilot data in this model, anticipating ~40% treatment effect with SD ≈ 20% of the mean. This was estimated to give ~80% power (β = 0.20) at α = 0.05 for detecting differences in salivary flow, while adhering to reduction in animal use. Post-hoc power analyses were performed using observed salivary flow means and SDs. For the three-group comparison (one-way ANOVA, fixed effects, α = 0.05), the calculated effect size was 2.29, yielding 100% achieved power. For the RT versus sham-irradiated contrast (two-tailed t-test, α = 0.05), Cohen’s *d* was 5.20, also corresponding to 100% power. These values confirm that the chosen sample size was adequate for detecting the observed differences.

## 3. Results

### 3.1. Radiation-Induced Apoptosis in Endothelial Cells Is Prevented by Pretreatment with NACA

**Figure 1:**
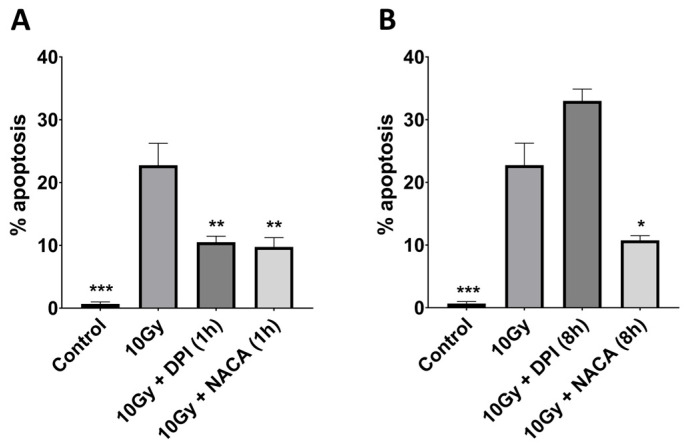
Bovine aortic endothelial cell apoptosis 1 h (**A**) and 8 h (**B**) following radiation. DPI and NACA were added to the cells 10 min prior to 10 Gy irradiation (10 µM and 1 mM, respectively). * *p* < 0.05; ** *p* < 0.001; *** *p* < 0.0001. DPI, diphenyleneiodonium chloride; NACA, N-acetylcysteine amide.

To examine the radioprotective potential of NACA, BAECs were pretreated with either NACA (1 mM) or DPI (10 µM), a known NADPH oxidase inhibitor, 10 min prior to irradiation. Both compounds demonstrated significant protective effects when apoptosis was assessed 1 h after exposure to radiation. Specifically, NACA and DPI reduced the rate of apoptosis by 54% and 57%, respectively (*p* < 0.01) (Figure 1A).

To assess whether prolonged exposure would influence these protective effects, the experiment was extended to include an 8 h post-irradiation time point. Interestingly, under these conditions, DPI treatment increased apoptosis by 45%, while NACA maintained its protective effect, reducing apoptosis by 53% at 8 h (*p* < 0.05) (Figure 1B).

**Figure 2:**
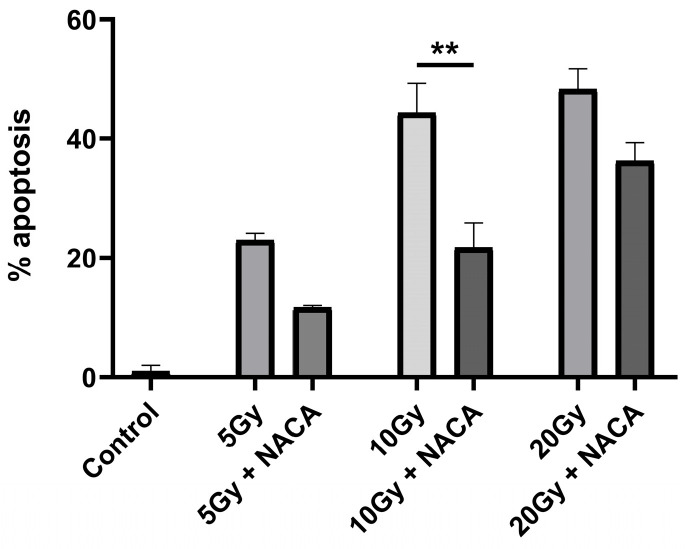
Endothelial cell apoptosis in response to increasing doses of radiation. NACA (1 mM) was added to the cells 10 min prior to 5, 10, and 20 Gy irradiation. ** *p* < 0.001.

In a dose–response study, the ability of NACA to counteract apoptosis across a range of radiation doses was evaluated. BAECs were exposed to increasing doses of ionizing radiation to determine the dose-dependence of NACA’s protective effect. As shown in Figure 2, NACA significantly inhibited apoptosis at all radiation doses tested, although to a lesser extent as the radiation doses increased. Additionally, a comparison of 1 mM versus 5 mM concentrations of NACA revealed no significant additional benefit at the higher dose, indicating that 1 mM may be sufficient to achieve maximal protection under these conditions.

### 3.2. NACA Is Effective Against Long-Term Effects of Radiation on SG Microvasculature

**Figure 3:**
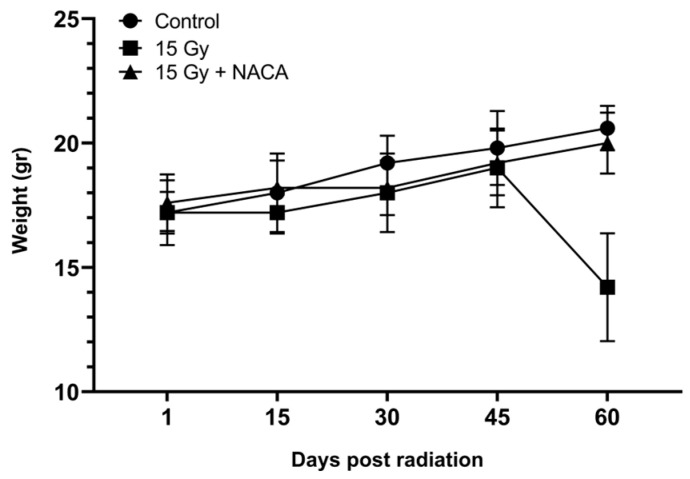
Average body weight of mice during 8 weeks after treatment with 0 or 15 Gy radiation only or 15 Gy radiation with NACA 350 mg/kg (N = 5 mice per group).

**Figure 4:**
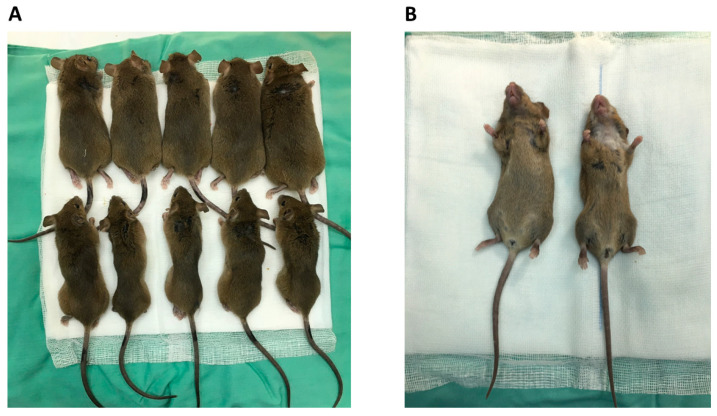
NACA-treated mice ((**A**)—top row; (**B**)—left side) compared to the untreated irradiated group ((**A**)—bottom row; (**B**)—right side).

Eight weeks following radiation treatment, untreated irradiated C3H mice exhibited a marked decline in general health and lost a significant amount of body weight, as shown in Figure 3. In addition to weight loss, they developed pronounced alopecia in the head and neck regions, the targeted sites of irradiation. Visual assessment revealed poor grooming and lethargic behavior, which contributed to an overall impression of decreased well-being and failure to thrive in the weeks following RT (Figure 4A, bottom panel; Figure 4B, right side). In contrast, mice that were pretreated with NACA showed not only a complete prevention of weight loss but actually gained weight over the same period, as shown in Figure 3. These animals also retained their fur in the irradiated region, without visible signs of skin irritation or alopecia. Their external appearance and physical activity remained similar to sham-irradiated control mice throughout the observation period (Figure 4A, upper panel; Figure 4B, left side).

**Figure 5:**
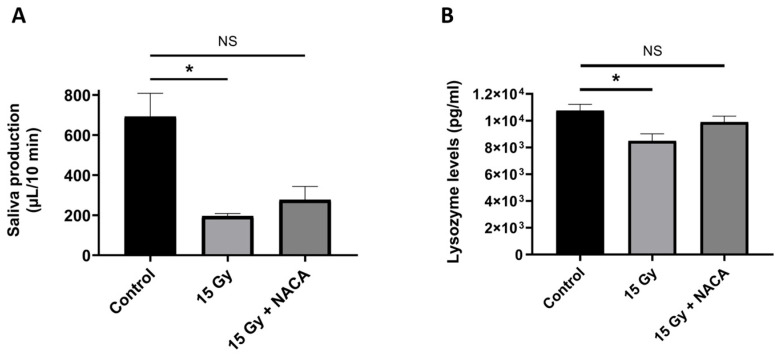
(**A**) Average saliva production collected during 10 min after subcutaneous administration of pilocarpine 1 µL/g body weight (50 mg/mL) in mice 8 weeks after treatment with 0 or 15 Gy radiation only or 15 Gy radiation with NACA 350 mg/kg (N = 5 mice per group). * *p* < 0.05 (**B**) Average saliva lysozyme concentration measured in mouse saliva 8 weeks after treatment with 0 or 15 Gy radiation only or 15 Gy radiation with NACA 350 mg/kg using ELISA (N = 5 mice per group). * *p* < 0.05. NS: not significant.

Using a pilocarpine stimulation assay, we evaluated salivary flow as a functional biomarker for SG hypofunction. Untreated irradiated mice demonstrated a marked reduction in salivary output, consistent with the development of radiation-induced salivary hypofunction. Specifically, the average salivary flow in these irradiated animals was significantly decreased, measuring 195 ± 12.7 μL/10 min, in contrast to the sham-irradiated control group, which exhibited a substantially higher salivary flow rate of 692 ± 116.5 μL/10 min (*p* = 0.0237). In contrast, mice treated with NACA following irradiation exhibited a partially preserved salivary output. The salivary flow in NACA-treated animals was 277 ± 67 μL/10 min, a value that did not differ significantly from the sham-irradiated control group (*p* = 0.056) (Figure 5A). There was no statistically significant difference observed in the direct comparison between the RT and RT + NACA groups (p > 0.999). Post-hoc power analysis indicated 100% power for the three-group ANOVA and 100% for the RT vs. sham-irradiated comparison. In addition to flow rate measurements, we also quantified lysozyme levels in the saliva as a surrogate marker for secretory protein output and glandular health. A corresponding trend was observed in this parameter as well. Lysozyme levels followed a similar pattern to salivary flow, suggesting a protective effect in the NACA-treated group (*p* = 0.0325) (Figure 5B). No statistically significant difference was observed in direct comparison between RT and RT + NACA (*p* = 0.311).

**Figure 6:**
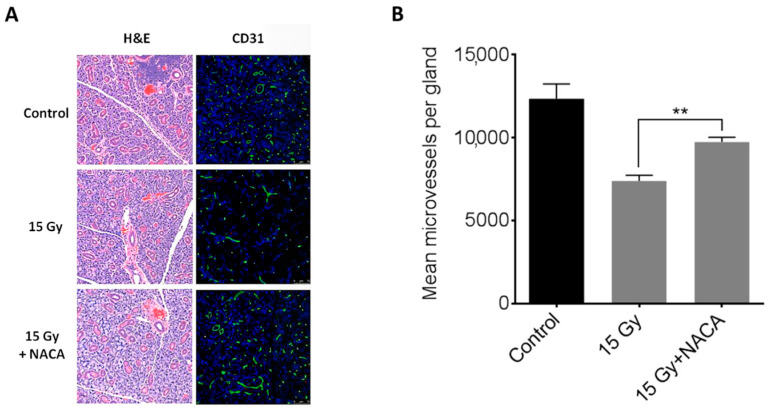
(**A**) Representative H&E and immunofluorescent staining with anti-CD31 antibody of mouse salivary glands 8 weeks after treatment with 0 or 15 Gy radiation only or 15 Gy radiation with NACA 350 mg/kg. CD31-positive vessels appear in green and nuclei in blue (scale bar = 75 µm). (**B**) Microvessel density (MVD) eight weeks following radiation. MVD in salivary glands was calculated as the number of CD31 + area per gland from four different sections per mouse (N = 5 mice per group). ** *p* < 0.001.

While no apparent gross histological differences were noted between the SG of irradiated and nonirradiated mice upon routine H&E staining, a more detailed quantitative analysis revealed significant alterations in the microvascular architecture. Specifically, MVD within the SG was markedly reduced in the untreated irradiated group compared to the nonirradiated control animals. When NACA was administered prior to radiation exposure, this decline in MVD was significantly attenuated. The MVD levels in the NACA-treated irradiated group were comparable to those observed in the control group (*p* < 0.001) (Figure 6A,B).

## 4. Discussion

In the current study, we investigated the potential role of the newly synthesized antioxidant compound N-acetylcysteine amide as a radioprotective agent specifically targeting salivary gland function during and after radiation exposure. Our approach consisted of both in vitro and in vivo models to provide a comprehensive evaluation. Initially, we demonstrated that ROS scavengers can protect endothelial cells from undergoing apoptosis following radiation exposure. This finding supports the previously described mechanism in which ceramide and ROS mediate microvascular dysfunction, a pathway extensively reported in earlier studies [10,11,12]. These early results formed the basis for our subsequent investigation of NACA’s protective efficacy in an in vivo setting.

Several prior studies from our group have focused on understanding the adverse effects of radiation on the microvascular environment within the SGs, with particular emphasis on endothelial cell damage. Based on this body of work, we proposed a model in which radiation-induced microvascular dysfunction is primarily driven by activation of acid sphingomyelinase, resulting in the generation of ceramide-rich macrodomains. This in turn leads to the recruitment and activation of NADPH oxidase, thereby promoting the generation of ROS [22]. These redox signaling platforms disrupt vascular integrity and impair tissue perfusion. Supporting this model, Li et al. described a similar mechanism wherein the ASMase/Ceramide pathway contributes to microvascular injury by organizing the membrane into redox-signaling platforms via NADPH oxidase recruitment [23]. Furthermore, in a recently published study from our group, we demonstrated that preincubation of endothelial cells with N-acetylcysteine inhibited radiation-induced ceramide accumulation, suggesting that the generation of ceramide is ROS-dependent and not merely a direct response to radiation [12].

Although NAC and other thiol-based antioxidants have been studied extensively as potential radioprotectors, their clinical application has been limited primarily by issues related to systemic toxicity, poor bioavailability, and limited cellular penetration. In contrast, NACA has been shown to effectively penetrate cell membranes and replenish intracellular stores of reduced glutathione by participating in cellular redox homeostasis. Through these mechanisms, NACA is able to confer protection against oxidative damage [24]. While a number of studies have investigated the role of NACA in disease models associated with oxidative stress, data regarding its role as a radioprotective agent, particularly in the context of salivary gland preservation, are limited. In one notable study, Wu et al. examined the effects of NACA on irradiated Chinese hamster ovary cells and reported that NACA attenuated radiation-induced cytotoxicity in a dose-dependent fashion, largely through restoration of reduced glutathione levels. Notably, they also reported a more favorable toxicity profile for NACA compared to NAC, further supporting its potential clinical utility [25]. In the current study, we found that NACA administration reduced radiation-induced endothelial cell apoptosis by over 50%, demonstrating potent cytoprotective activity. Moreover, when compared to the NADPH oxidase inhibitor DPI, NACA was found to be equally effective in mitigating radiation-induced apoptosis in endothelial cells. However, a key distinction was noted in toxicity profiles: extended exposure to DPI resulted in significant cytotoxicity, whereas NACA continued to exhibit radioprotective effects without any detectable increase in toxicity, even when cells were exposed for up to 8 h post-radiation. These findings not only highlight the efficacy of NACA as a ROS scavenger but also underscore its low toxicity, an essential requirement for any compound intended for therapeutic use. Notably, the protective effects of NACA were sustained even at higher doses of radiation, suggesting that it could be effective in more aggressive treatment regimens.

To build on these in vitro results, we evaluated the in vivo impact of radiation on salivary gland function using a murine model and further assessed the ability of NACA to mitigate these effects. In line with clinical observations in patients receiving radiotherapy for head and neck squamous cell carcinoma, we found that a single 15 Gy dose of radiation delivered to the orthotopic head and neck region in mice led to measurable clinical and biochemical deterioration of SG function. These effects included significant weight loss, reduced appetite, and decreased salivary flow rates, as well as reductions in lysozyme levels. Notably, weight loss emerged after a latency of approximately 2 months, consistent with prior rodent studies such as Nagler et al., which reported delayed, dose-dependent systemic decline and weight loss following head and neck irradiation [26]. These functional impairments were accompanied by histopathological evidence of microvascular injury within the SGs. Notably, pretreatment with NACA attenuated these radiation-induced effects. In addition to preserving salivary flow and lysozyme output, NACA conferred protection against radiation-induced alopecia, suggesting a potential role as a systemic ROS scavenger. Interestingly, while other antioxidants such as NAC and Tempol failed to prevent the post-radiation decline in lysozyme levels [12], NACA succeeded, suggesting a broader mechanism of action. Whether this broader protection is due to additional, yet uncharacterized, effects on saliva composition or epithelial function remains an area for future investigation [27]. Histological analyses further demonstrated a decrease in tissue perfusion within the SGs following irradiation, likely due to microvascular damage. Pretreatment with NACA significantly ameliorated this vascular compromise, suggesting that preservation of the microvasculature is a key mechanism through which NACA maintains salivary gland function post-irradiation. These findings are particularly relevant given the central role of microvascular integrity in glandular regeneration and fluid secretion.

One of the greatest challenges in delivering effective radiation therapy for head and neck cancer lies in achieving maximal tumor control while minimizing collateral damage to normal tissues. This has long motivated the search for selective radioprotectors that can preserve non-malignant tissue without protecting the tumor. Amifostine, currently the only FDA-approved agent for the prevention of xerostomia in patients undergoing RT for HNSCC, has seen limited adoption due to concerns over potential tumor protection. Some studies have suggested that Amifostine may reduce the therapeutic efficacy of radiation by decreasing tumor radiosensitivity [28]. In contrast, other agents such as Tempol have been proposed as selective radioprotectors that spare salivary glands while not protecting tumor tissue, likely due to more rapid metabolic conversion to non-protective derivatives in tumors compared to normal tissues [29,30,31].

In our current study, NACA demonstrated protection of SG function following irradiation; however, several important limitations should be acknowledged. First, we used healthy, non–tumor-bearing mice as an initial proof-of-concept model to isolate treatment effects on normal tissue; therefore, the broader impact of NACA on tumor radiosensitivity remains unknown. Second, only female mice were included in this preliminary work to reduce cage aggression and achieve more homogeneous cohorts; however, sex-related differences in salivary gland biology and radiation response may limit generalizability to males. Third, although our data are consistent with inhibition of ROS-driven ASMase/ceramide signaling, we did not directly quantify ceramide levels, ROS markers, or apoptotic indices in vivo. Together, these considerations highlight the need for studies in tumor-bearing models with sex-balanced cohorts and direct biochemical evaluation of the ASMase/ceramide–ROS pathway to fully establish the therapeutic potential and clinical translatability of NACA as a radioprotective agent. Our group is actively undertaking such investigations using an orthotopic sex-balanced HNSCC model to assess NACA’s radioprotective profile in a clinically relevant context. Further research will determine whether cytoprotective dosing confers any tumor protection and how this may affect NACA’s safety and efficacy as a radioprotector.

## 5. Conclusions

RT-induced microvascular dysfunction leads to SG hypofunction, an effect that is significantly attenuated by pretreatment with NACA. These findings strongly suggest that NACA may serve as a potential radioprotector of salivary gland function in patients undergoing radiation therapy for HNSCC.

## Data Availability

Data is contained within the article.
